# Implementation of a knowledge mobilization model to prevent peripheral venous catheter-related adverse events: PREBACP study—a multicenter cluster-randomized trial protocol

**DOI:** 10.1186/s13012-018-0792-z

**Published:** 2018-07-25

**Authors:** Ian Blanco-Mavillard, Miquel Bennasar-Veny, Joan Ernest De Pedro-Gómez, Ana Belén Moya-Suarez, Gaizka Parra-Garcia, Miguel Ángel Rodríguez-Calero, Enrique Castro-Sánchez, on behalf of the Research Group PREBACP, Luis Moreno-Mejías, Cynthia Sánchez-Rojas, Luis Molero-Ballester, Ismael Fernández-Fernández, Araceli Prieto-Alomar, Francisco Ferrer-Cruz, Josefa Cardona-Rosello, Sara Zaforas-Sánchez, Gràcia Mut-Salvà, Vanesa Gómez-Queipo

**Affiliations:** 1 Hospital de Manacor, Quality, Teaching and Research Unit, Manacor, Spain; 2 Evidence, Lifestyles and Health Research Group, Research Institute of Health Sciences, Universitat de les Illes Balears, Palma, Spain; 3 Department of Nursing and Physiotherapy, Universitat de les Illes Balears, Palma, Spain; 4 Department of Nursing, Agencia Sanitaria Costa del Sol, Marbella, Málaga, Spain; 5 Hospital San Juan de Deu, Palma, Spain; 6 Health Care Office, Balearic Islands Health Service, Palma, Spain; 7 NIHR Health Protection Research Unit in Healthcare Associated Infection and Antimicrobial Resistance at Imperial College London, London, UK

**Keywords:** Knowledge mobilization, Evidence-based practice, Clinical practice guideline, Catheter- related adverse events, Peripheral venous catheterization

## Abstract

**Background:**

Peripheral venous catheters are the most commonly used invasive devices in hospitals worldwide. Patients can experience multiple adverse events during the insertion, maintenance, and management of these devices. Health professionals aim to resolve the challenges of care variability in the use of peripheral venous catheter through adherence to clinical practice guidelines. The aim of this cluster-randomized controlled trial is to determine the efficacy of a multimodal intervention on incidence of adverse events associated with the use of peripheral venous catheters in adult hospital patients. Additional aims are to analyze the fidelity of nurses and the relationship between contextual factors on the use of best available and the outcomes of the intervention.

**Methods:**

Five public hospitals in the Spanish National Health System, with diverse profiles, including one university hospital and four second-level hospitals, will be included. In total, 20 hospitalization wards will be randomized for this study by ward to one of two groups. Those in the first group receive an intervention that lasts 12 months implementing evidence-based practice in healthcare related to peripheral catheters through a multimodal strategy, which will contain updated and poster protocols insertion, maintenance and removal of peripheral venous catheters, technologies applied to e-learning, feedback on the results, user and family information related to peripheral catheter, and facilitation of the best evidence by face-to-face training session. Primary outcome measures: Incidence of adverse events associated with the use of peripheral venous catheters is measured by assessing hospital records. Secondary outcome measures: Nurses’ adherence to clinical practice guidelines, clinical outcomes, and the cost of implementing the multimodal intervention.

**Discussion:**

Clinical implementation is a complex, multifaceted phenomenon which requires a deep understanding of decision-making, knowledge mobilization, and sense making in routine clinical practice. Likewise, the inclusion of strategies that promote fidelity to recommendations through multicomponent and multimodal intervention must be encouraged. The use of a transfer model could counterbalance one of the greatest challenges for organizations, the evaluation of the impact of the implementation of evidence in the professional context through quality indicators associated with prevention and control of infections.

**Trial registration:**

Current Controlled Trials ISRCTN10438530. Registered 20 March 2018.

## Background

Peripheral venous catheters (PVC) are the most commonly used invasive devices in hospitals worldwide [1]. Patients can experience multiple adverse events such as phlebitis, extravasation, or infections during the insertion, maintenance, and management of these devices [2]. Among these adverse events, catheter-related bloodstream infections (CRBSI) are catastrophic [3] yet potentially preventable episodes [4]. The incidence of PVC-associated bloodstream infections (PVC-BSI) is between 0.1 and 0.5 per 1000 catheter days [5]. CRBSIs can prolong length of hospital stay [2, 6] and carry an attributable mortality rate of up to 25% [7, 8]. The approximate average cost per episode of CRBSI is $45,000 and thus resulting in $2.3 billion of unnecessary expenditure per year in the USA [3].

The genesis of evidence-based clinical practice (EBCP) is the integration of best available scientific knowledge in combination with clinical experience and user preferences on health and care issues [9–11]. Clinical practice guidelines (CPGs) are based on empirical evidence, developed critically with explicit methods by experts, free of conflicts of interest and with specific and unambiguous recommendations [12]. These guidelines are developed to assist the decision-making of health professionals and patients about appropriate health care interventions in specific clinical circumstances. However, CPGs are not exempt from challenges [13]. The number of guidelines has grown significantly and thus the volume of evidence proven to be unmanageable and of variable quality [14]. Additionally, there is frequent tardiness in the implementation of the recommendations within the CPGs, probably fueled by perceptions of clinical judgment as the main element in clinical decision-making [15]. These facts can ultimately weaken the credibility of CPGs and therefore increase the difficulty of their implementation [16].

In the last decade, healthcare systems have also focused on reducing the variability of healthcare practice [17]. International research agencies have conducted strategies to effectively implement knowledge to resolve the challenges presented by clinical practice variability and offer optimal, quality care to patients and citizens [18].

However, the introduction of innovations into daily clinical practice remains arduous. Despite efforts to reduce the research-practice gap, some studies suggest that 30–40% of patients are still not offered care based on best available evidence [19, 20]. Such gap is therefore a major threat to patient safety and healthcare efficiency [21]. The use of implementation models aims to enable the integration of key elements which are in permanent and dynamic interaction, such as research result innovations, the individuals and teams that have to enact the change and local and organizational context, which will be supported through the process of facilitation to warrant effective knowledge mobilization (EKM) [22–27]. Therefore, the incorporation of a knowledge mobilization model could be a feasible approach to reduce such research-practice gap that it embed a deep understanding of decision-making and key elements to promote adherence of evidence-based practice [28].

This protocol describes a theoretical model to evaluate the effectiveness of a multimodal intervention focused on implementing evidence into clinical practice. Drawing from the core elements of evidence, context, and facilitation present on The Promoting Action on Research Implementation in Health Services (PARIHS) framework, we will determine the efficacy of our planned intervention on the incidence of adverse events associated with the use of PVC in adult patients in hospital. This research will also unpack the relationship between factors influencing local context and individual perceptions about the use of evidence-based practice.

## Methods/design

### Aims

1. To determine the efficacy of a multimodal intervention to reduce the incidence of adverse events (CRBSI, extravasation, obstruction, and phlebitis) associated with the use of PVC in adult patients in hospital.
2. To analyze the fidelity of nurses to the recommendations within the CPG for insertion and management of PVCs.
3. To associate post-intervention adverse event rates with contextual and individual factors on the use of best available knowledge in clinical practice decisions.

### Primary hypothesis

The implementation of a multimodal intervention will decrease the incidence of adverse events (CRBSI, extravasation, obstruction and phlebitis) associated with the use of PVCs in adult patients in hospital.

### Secondary hypotheses

1. Nursing practice outcomes: An optimal fidelity of nurses to the recommendations within the CPG for insertion and management of peripheral venous catheter in hospital wards receiving the intervention will translate into reduced care variability, increased documentation about PVC use in nursing records and greater requests of catheter tip culture from PVCs removed from patients experiencing adverse events.
2. Clinical outcomes: The fidelity of nurses to the recommendations within the CPG for insertion and management of peripheral venous catheter in hospital wards receiving the intervention will reduce unnecessary PVCs and decrease hospital length of stay (HLOS).
3. Health economic outcomes: The implementation and development costs of the intervention will be offset by savings from decreased incidence of CRBSIs and HLOS.
4. EBCP environment: The contextual and individual factors on the utilization of knowledge in clinical practice decisions and impact on hospital ward processes and practice measured by Nursing Work Index (NWI) [29] and Evidence-Based Practice Questionnaire (EBPQ) [30].

### Design

This knowledge mobilization study uses a pragmatic cluster-randomized controlled trial (C-RCT) design, with embedded process evaluation. Such design will allow the measurement of clinical efficacy and costs of a multimodal intervention to improve PVC-related adverse events as such as PVC-BSI and phlebitis. We will compare outcomes and costs from implementation of CPGs. The embedded process evaluation will elicit the variable contexts of implementation, the barriers and enablers encountered, the response by stakeholders, and the resources required for implementation. The multicenter nature of the study, with the inclusion of hospitals with different organizational characteristics and located in different geographic areas, will enhance the diversity of the sample and thus its external validity. Reporting of this trial will adhere to the CONSORT statement and its extension to C-RCTs [31].

### Setting

The study will be conducted in five public hospitals with diverse characteristics within the Spanish National Health System, including one reference hospital and four acute care hospitals. Twenty wards will be selected and randomly allocated to either the intervention or control groups. The intervention will be delivered at ward level, and therefore, the ward will be considered the unit of analysis. Emergency, critical care, pediatric, maternity, peri-operative, operative rooms, and psychiatric areas will be excluded from the analysis, due to the fact that peripheral catheters are routinely maintained inserted for less than 24 h. Bias-compensating measures will be incorporated to homogenize nursing practice through face-to-face training in excluded wards.

### Sample/participants

All healthcare staff working on the study wards and delivering direct care to adult inpatients will be involved in the study. To ensure homogeneity between units, each ward enrolled in the study must have a stable permanent staff, reducing the possibility of contamination by personnel movement.

### Primary outcome measures: effect evaluation

The primary outcome will be the incidence of adverse events associated with the use of PVCs in adult inpatients. This incidence will be determined from evaluation of hospital records at 3, 6, 9, and 12 months.

### Secondary outcome measures: process evaluation

1. Nurses’ adherence to CPGs will be measured at 3, 6, 9, and 12 months with the following subcategories:
  1.1 Multimodal intervention content will be delivered as planned (yes/no), and dosage will be delivered as often and long as planned (yes/no); face-to-face training session will be assessed by measuring the number of nurses who completed the Masterclass at the intervention phase. Feedback will be evaluated by checking the distribution of clinical audit results within intervention wards. Patient information will be assessed by monitoring the presence of informative leaflets on the ward (yes/no) and asking if the patient is aware of use of PVC and prevention recommendations for personal care. Facilitation will be measured by number of internal facilitators who completed the intervention.
2. Clinical audits on use of PVC. Device utilization ratios will be measured by percentage of PVCs per hospital wards and number of PVC per patient. Documentation of PVC in nursing records will be assessed by percentage of fully completed records. PVC maintenance will be monitored by random, monthly clinical audits, which will document PVC size (16/18/20/22/24 gauge), site (dorsum of hand/forearm/antecubital region/upper arm), dressing integrity (clean/dry/intact), securement and time in situ (less 48 h/ between 48 and 96 h/more 96 h). Catheter tip will be measured by number of catheter tip extraction following removal of PVCs.
3. Routine practice. Clinical effectiveness questionnaire for the prevention of PVC complications will be completed pre and post intervention. The questionnaire is made up of 35 questions in four sections, relating to general asepsis and skin antisepsis; PVC insertion, maintenance, and removal; PVC documentation; and patient and professional education.
4. Clinical outcomes will be assessed using rates of unnecessary PVCs, rates of CRBSI, extravasation, obstruction and phlebitis, defined as per standard guidelines, and mean HLOS associated to PVC-BSIs at 3, 6, 9, and 12 months.
5. The direct costs of implementing the multimodal intervention will be assessed using HLOS for patients and decrease rates of adverse events at 12 months (post intervention).
6. EBCP environment:
7. EBCP context: The contextual factors on the utilization of knowledge in clinical practice decisions and impact on hospital ward processes and practice will be assessed using the NWI tool pre-intervention. The variables are summarized into five main groups: nurses’ participation in hospital affairs; quality of nursing care; nurse management’s capacity, leadership, and support for nursing staff; size of the nursing workforce and adaptation of available human resources, and professional relation between doctors and nurses.
8. EBCP individual: The opinions, attitudes, abilities and motivations of nurses and their links with the development of a culture of clinical practice based on the transfer of new knowledge to the healthcare given to patients will be assessed using EBPQ pre-intervention. The questionnaire is made up of 24 questions relating to professionals’ knowledge, use and attitudes towards EBCP.

### Sample size

Calculations are based on a previous observational pilot conducted in Manacor Hospital, which reported a global rate of 44.1% PVC-associated adverse events (16% phlebitis, 6.8% obstruction, 18.1% extravasation, and 3.2% CRBSIs rates of which 3.1% were CRBSIs type 1, 0.14% CRBSIs type 2, and none CRBSIs type 3). Similar studies have a potential for improvement between 7 and 19% in adverse event rates, such as phlebitis, infiltration, and obstruction, yet there is no statistical significance or potential for improvement in CRBSI rates [32, 33]. The initial assumption is for the intervention to decrease the rate of adverse events associated with PVC in the intervention group at 6 months post-intervention by 15%. For such target result, the sample size required would be 1920 nursing records, accepting an alpha risk of 0.05, a beta risk of 0.2 in a two-sided test, and 10% missing data. For the calculation of sample size, the smallest detectable difference with adequate power and statistical significance has been considered. The sample size has been corrected to account for within-intra cluster correlation coefficient of 0.01 to allow for a design effect of 1.99. The final sample size will therefore be of 3821 nursing records, assuming an average cluster size of 200 nursing records.

### Control group

The control ward will not receive the multimodal intervention and will continue with routine practice.

### Intervention group

**Fig. 1:**
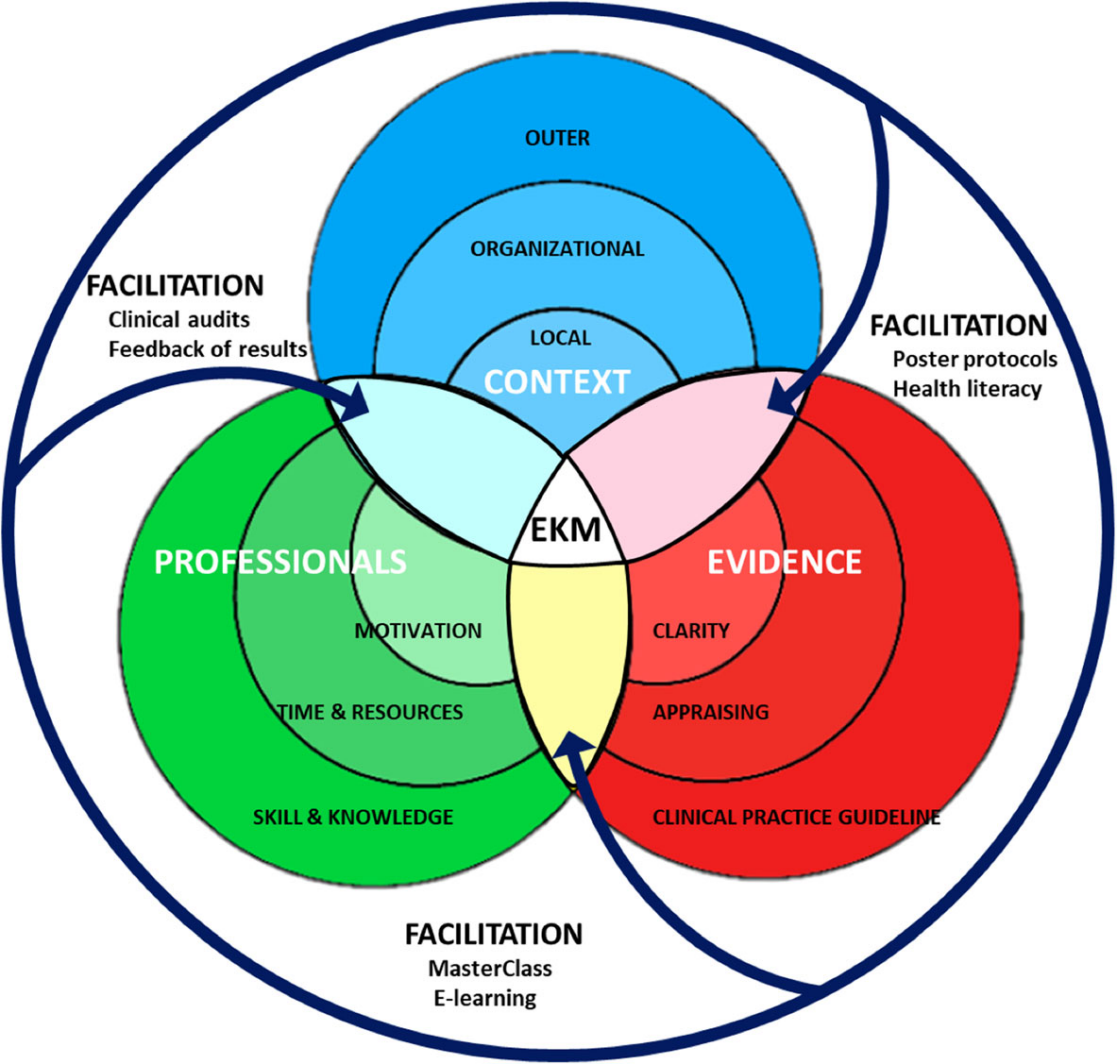
Theoretical model on effective knowledge mobilization

The intervention will last 12 months and will be based on a theoretical model on effective knowledge mobilization, integrating a multimodal strategy related to peripheral catheters clinical practice improvement which includes the following (Fig. 1): (1) implementation of recommendations trough up-to-date protocols and posters related to hand hygiene and aseptic measures, insertion, maintenance and removal of PVC [34]; (2) use of e-learning technologies [35–37]; (3) feedback on the results and messages addressed to healthcare professionals to facilitate adherence to recommendations [38]; (4) face-to-face training sessions [39]. Masterclass related to PVC insertion, maintenance, and removal will consist of information shared about recommendation of CPGs adapted according to the needs detected by means of the questionnaire on the effectiveness of the healthcare practice; (5) leaflets with information for patients and family/careers about peripheral catheters, in appropriate language [40]; and (6) support by internal facilitators, which will be key members of staff in the organizations, to adopt best evidence based on the PARIHS theoretical model [26, 41, 42].

This facilitation will be carried out by nurses using the Facilitating Implementation of Research Evidence (FIRE) approach, and who will flexibly tailor implementation strategies to the local ward context, and to resolve barriers and enablers identified. There will be two types of FIRE agents: hospital leaders (hFIRE) and hospital ward nurses (nFIRE). These agents will be allocated to the wards of each hospital. To mitigate the potential risk of facilitators leaving their posts during the study period we will deploy a co-facilitation model with three support nurses on each intervention ward. nFIRE nurses will be appointed to support and train other nurses on the GPC recommendations, carrying out the face-to-face training in their units, working in small groups to review process indicators and routines practices. Both nFIRE and hFIRE will lead an education program based on the theory of planned behavior [43–45] which includes behavioral techniques to facilitate the application of evidence-based practice. At the hospital level, the hFIREs will audit PVC-related practices, identifying barriers and providing support and guidance to resolve such hurdles. The facilitation strategy will be led by expert external facilitators, PREBACP group research, working with both types of FIRE.

### Data collection

To mitigate control bias, each nurse manager will be provided with information to standardize catheter removal, catheter tip culture, and hemoculture extraction. Tips from all PVC removed from patients experiencing adverse events will be cultured using a semiquantitative method. Clinical, microbiological, and ward information will be collected from each patient on PVC removal.

**Fig. 2:**
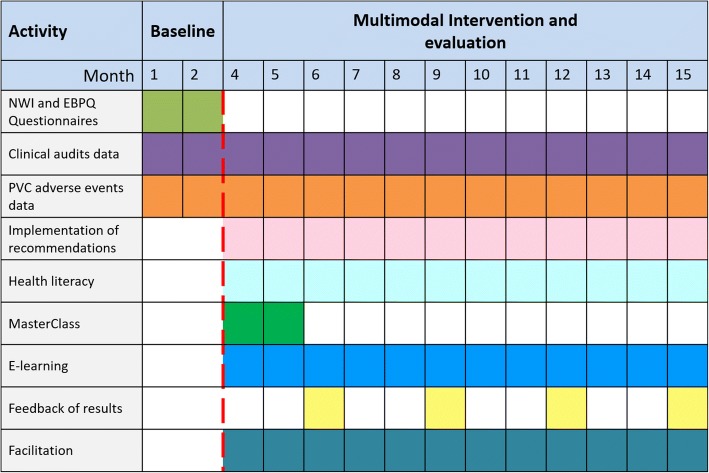
Timeline of PREBACP study

Project investigators will collect primary and secondary outcome data using a wide range of methods, including questionnaires NWI and EBPQ to nurses, clinical audits monthly, and clinical outcome by requesting statistics report the participating wards at each hospital with the variables. Where these data are not available, clinical outcome data will be adapted from our research to collect the primary and secondary outcome data. Tools will be used for communication between facilitators, auditors, and researchers to collect at the time of activity and throughout the duration of the intervention. The mean hospital length of stay for relevant wards will be collected as reported by the hospital electronic patient management systems. This data collection system was piloted in five of the above hospital units in December 2017. Adverse events will be defined as per international guidelines for the prevention of BSIs (Healthcare Infection Control Practices Advisory Committee, USA, UK, Spain) [46–48]. Three study phases will be planned for the PREBACP study: baseline (2 months), intervention and evaluation (12 months) (Fig. 2).

### Definitions

- CRSBI: The following case definitions will be used to determine a diagnosis of CRBSI (see Fig. 1):

- ▪CRBSI type 1: Local PVC-related infection (no positive blood culture): Positive quantitative culture (103 CFU/ml) or semi-quantitative culture with more than 15 CFU from the tip of PVC and local signs of infection at the insertion site or in catheter lumen.
- ▪CRBSI type 2: General PVC-related infection (no positive blood culture positive): positive quantitative culture (103 CFU/ml) or semi-quantitative culture with more than 15 CFU from the tip of PVC and that clinical signs improve within 48 h of catheter removal.
- ▪CRBSI type 3: PVC-BSI associated with microbiologically confirmed with blood culture occurring 48 h before or after catheter removal and positive quantitative culture (103 CFU/ml) or a semi-quantitative culture with more than 15 CFU from the tip of PVC for the same microorganism.

(b) Phlebitis: An inflammation of the wall of a vein. If a blood clot in the vein causes the inflammation, then the condition is termed thrombophlebitis. This problem is characterized by persistent pain referred to PVC (2 h since the last administration), erythema, swelling, and palpable thrombosis of the cannulated vein.
(c) Extravasation: Inadvertent leakage of a vesicant solution into surrounding tissue.
(d) Obstruction: Following occlusion of the PVC, which can be partial (i.e., blood cannot be aspirated, but PVC can be flushed) or complete, whereby neither aspiration nor infusion are possible.

### Data analysis

#### Effect evaluation and process evaluation

Quantitative methods will be used to analyze nursing practices, health service utilization, and economic outcomes. Main and secondary outcome analyses will be based on all randomized wards and selected participants. To account for within-patient correlation, due to multiple measurements from the same patient during assessment days, we will implement generalized estimating equation models with binary outcome and logic link for all rate outcome comparisons. The statistical analysis will consist of an exploration of the descriptive data of the sample, bivariate analysis with parametric and non-parametric tests, depending on the nature of the distributions (correlation, ANOVA, chi-square) and multivariate (multiple regression with independence analysis using Durbin-Watson statistics). The Cochran-Mantel-Haenszel test will be used to compare proportions. A series of exploratory analyses will be conducted on sub-groups and the impact of covariates on estimated of the effects of the intervention. A nonparametric median test will be used for HLOS comparison. In supportive analysis, HLOS will be considered as time to event data. Survival rates will be calculated and illustrated by the Kaplan-Meier method and further analyzed by the long rank test for univariate analysis. Variables that reveal prognostic or effect modifying potential on the outcome as suggested by univariate analysis will subsequently be evaluated by the proportional Cox regression for multivariate analysis. Hazard ratios with corresponding 95% confidence intervals will be reported. A *p* value of < 0.05 will be considered statistically significant. A regression model will be constructed to further explore the results obtained in the bivariate analysis, seeking to establish an explanatory model on the variables involved in improving the use of recommendations. Data will be analyzed using the program SPSS IBM Statistics version 21.

#### BCP environment

Firstly, an exploratory analysis will be performed of characteristics that define the behavior of each of the variables used, by means of classical descriptive techniques and the exploratory data analysis procedure. Secondly, relations will be analyzed between the professionals’ answers to the two questionnaires (EBPQ and PES-NWI), bearing in mind the sociodemographic and occupational characteristics of the sample. Differential analyses will be conducted to generate specific profiles, using general linear model analysis techniques. The individual and contextual factors reported by the respondents will be modeled, taking into account personal and occupational characteristics and the hospital ward using a multi-level analysis.

### Randomization

Wards will be randomly allocated to the intervention or control arm using software, in blocks 1:1 with stratification by setting (medical or surgical) and hospital (to ensure homogeneity of both groups).

### Blinding

Hypothesis and variables will be blind to prevent any selection bias that might arise in the nurses participating in the data collection process. All research assistants will be blinded to group allocation. Success of blinding will be assessed at study end using the James Blinding Index. Although FIREs and intervention wards will not be blinded, control wards will be blinded. Patients will be unaware of the intervention. IBM and JDP analyze the data, all will be blinded to group allocation.

## Discussion

Clinical implementation is a complex, multifaceted phenomenon [26] which requires a deep understanding of decision-making, knowledge mobilization, and sense-making in routine clinical practice [49]. Likewise, the inclusion of strategies that promote fidelity [50] to recommendations through multicomponent and multimodal interventions [51, 52] must be encouraged. The identification of barriers and constraints at the level of institutions and individuals involved should be the first step [53]. The use of a transfer model could counterbalance one of the greatest challenges for organizations, the evaluation of the impact of the implementation of research evidence in the professional context through quality indicators associated with prevention and control of infections [54, 55]. Although life-threatening adverse events such as CRBSIs have a low incidence in our setting, the volume of PVC use amplifies its importance in terms of morbidity, mortality, and patient safety. An important limitation may be the low potential to reduce PVC-BSIs rates of through multimodal intervention [32, 56].

This protocol study will include the facilitation element based on the PARIHS framework, a key aspect with the potential to make substantial contributions to knowledge in this area [26, 41].

## Abbreviations

CPGs: Clinical practice guidelines
CRBSI: Catheter-related bloodstream infections
C-RCT: Cluster-randomized controlled trial
EBCP: Evidence-based clinical practice
EBPQ: Evidence-Based Practice Questionnaire
EKM: Effective knowledge mobilization
FIRE: Nurses facilitating implementation of research evidence
hFIRE: Hospital leaders
HLOS: Hospital length of stay
nFIRE: Hospital ward nurses
NWI: Nursing Work Index
PARIHS: Promoting Action on Research Implementation in Health Services
PVC-BSI: Bloodstream infections associated with PVCs
PVCs: Peripheral venous catheters
